# Population Genetics Reveals Insights Into *Cryophytum* Biogeography in South Africa

**DOI:** 10.1002/ece3.73667

**Published:** 2026-05-12

**Authors:** Clarke J. M. van Steenderen, Emma Sandenbergh, Iain D. Paterson

**Affiliations:** ^1^ Center for Biological Control, Department of Zoology and Entomology Rhodes University Makhanda Eastern Cape South Africa

**Keywords:** biogeography, Cape Fold Belt, hybridisation, ice plants, *Mesembryanthemum*, population genetics, RADseq

## Abstract

The Mesembryanthemoideae (Aizoaceae) are a diverse group of succulents in southern Africa, many of which are endemic to the Succulent Karoo and Cape Floristic Region of South Africa. This study investigated the population genetic structure and biogeography of two closely related species, *Cryophytum crystallinum* and *C. guerichianum,* and assessed whether abiotic factors, including temperature, precipitation and soil variables, contribute to their genetic differentiation. We also examined a putative hybrid from the Northern Cape Province, showing intermediate morphological traits. In *C. crystallinum* , populations at either end of the coastal range in the Western Cape Province were genetically similar, with distinct clusters occurring in between. We propose that repeated range shifts and the formation of refugia may explain this pattern and found little evidence that the abiotic variables considered here strongly shaped genetic structure. In *C. guerichianum*, we identified inland, intermediate and coastal genetic clusters, likely shaped by vicariance associated with the Cape Fold Belt. Across both species, genetic divergence showed some support for the Isolation by Environment hypothesis, with a correlation between genetic and environmental distances independent of geographic distance, although topography was likely the dominant driver. The suspected hybrids were genetically closer to *C. guerichianum*, suggesting asymmetric introgression and a possible mosaic pattern of hybridisation in areas of sympatry. As *C. crystallinum* is invasive in parts of the world, accounting for historical population structure and evolutionary history may help identify appropriate source populations for biological control, as genetically distinct populations can respond differently, and geographic distance alone may not reliably predict suitability.

## Graphical abstract

This study investigated the population genetic structure, biogeography and environmental drivers of divergence in Cryophytum crystallinum and C. guerichianum, two closely related Mesembryanthemoideae species, and evaluated a putative hybrid. Cryophytum crystallinum showed genetically similar populations at either end of its coastal range, with intermediate differentiation likely shaped by historical range shifts rather than the abiotic variables considered here. In contrast, C. guerichianum exhibited clear inland–coastal genetic structuring, most consistent with topographic isolation associated with the Cape Fold Belt, with some contribution from environmental gradients. Suspected hybrids were genetically closer to C. guerichianum, indicating possible asymmetric introgression. These findings have implications for the selection of source populations in biological control programmes targeting invasive *C. crystallinum* .

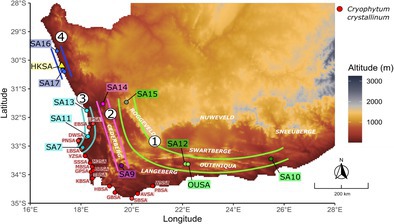

## 1 Introduction

The Mesembryanthemoideae subfamily (Aizoaceae) is almost exclusively endemic to southern Africa and is particularly diverse in the Succulent Karoo and Cape Floristic Regions (Chesselet 2004; Gerbaulet and Hartmann 2017). The group comprises at least 103 known species (Klak et al. 2014). Many genera within the Mesembryanthemoideae are known for their conspicuous epidermal bladder cells, which store water and salts, earning them the common name ‘ice plants’ (Gerbaulet and Hartmann 2017).

The taxonomy of the Mesembryanthemoideae has been debated in the literature. Klak et al. (2007), for example, proposed a single genus within the subfamily, *Mesembryanthemum*, while Gerbaulet (2012) advocated for genus‐level divisions. Since the *Mesembryanthemum* genus proposed by Klak et al. (2007) was confirmed to be polyphyletic, many of the former subgenera were reinstated (Gerbaulet and Hartmann 2017). Most Mesembryanthemoideae genera have since been revised, and the most up‐to‐date taxonomy for the group is presented in Gerbaulet and Hartmann (2017). The present work therefore refers to the aforementioned authority.

*Cryophytum crystallinum* L. and *C. guerichianum* Pax (formerly *Mesembryanthemum* subg. *Cryophytum*) are two closely related species indigenous to South Africa (Moran 1950; Adams et al. 1998; Gerbaulet and Hartmann 2017; Zaghloul et al. 2019). According to Gerbaulet and Hartmann (2017), *C. crystallinum* occurs from Namaqualand in the Northern Cape (NC) southward to the Cape Peninsula in the Western Cape (WC), and eastward toward Gqeberha (Port Elizabeth) in the Eastern Cape (EC). *Cryophytum guerichianum* occurs from Namaqualand, Kenhardt and Prieska in the NC, extending south to Malmesbury and Worcester in the WC, and east to Cradock in the EC. The two species do occur in sympatry in some parts of their distribution.

The current study used a RADseq population genetic approach to uncover structuring within *C. guerichianum* and *C. crystallinum* in their native distribution, and to investigate possible links in these patterns to biogeographical processes. We hypothesise that population structure has been influenced by abiotic factors (e.g., temperature, rainfall, soil characteristics and topography), supporting the ecological theory of ‘isolation by environment’ (IBE) (Wright 1943; Wang and Bradburd 2014). Although both species are opportunistic annuals associated with disturbed habitats, their establishment and survival are still influenced by abiotic factors to varying degrees. In the Cape Floristic Region (CFR), soil heterogeneity is well documented and is known to structure plant distributions (Goldblatt and Manning 2002; Verboom et al. 2012; Cowling and Potts 2015). We therefore considered soil‐associated environmental variation to be a plausible driver of population genetic structure, and a reasonable hypothesis to test given our genomic data and the availability of environmental layers.

Additionally, we sought to identify plant samples that displayed intermediate traits between the two *Cryophytum* species that were collected near Hondeklip Bay in the Northern Cape Province of South Africa. Better understanding the population genetics of *Cryophytum* taxa is important not only from a biodiversity perspective, but can also be valuable to invasion biology and biocontrol projects due to the invasive status of *C. crystallinum* in North America and Australia (van Steenderen et al. 2026).

## 2 Materials and Methods

### 2.1 Sample Collection

**FIGURE 1:**
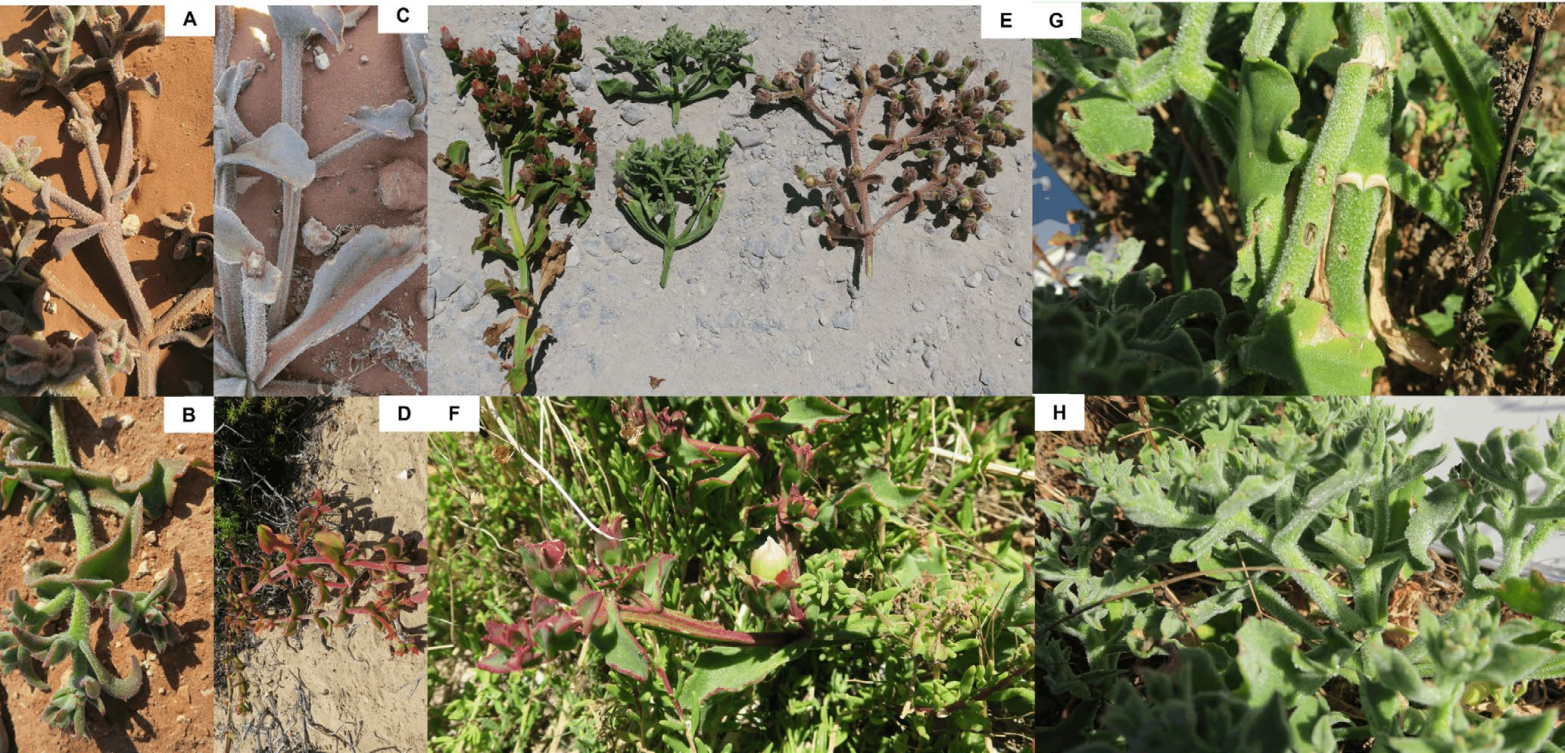
Morphological variation in stems among *Cryophytum* taxa and putative hybrids: (A) hybrid showing both round and square stems, (B) *C. crystallinum* with round stems, (C) *C. guerichianum* with winged to square stems, and (D) hybrid showing both round and square stems. (E) Comparison of *C. guerichianum* (left), *C. crystallinum* (middle), and a putative hybrid (right). (F) *C. guerichianum* exhibiting winged to square stems, (G) *C. crystallinum* with round stems and feeding damage, and (H) *C. crystallinum* with round stems. Photo credits: E. Sandenbergh.

Existing *Cryophytum crystallinum* RADseq data (NCBI Short Read Archive PRJNA1304995) were taken from van Steenderen et al. (2026), which included three specimens collected in Hondeklip Bay in the Northern Cape which displayed characteristics of both *C. crystallinum* and its close relative, *C. guerichianum* (Figure 1A–E). These two species are morphologically very similar, and the suspected hybrids could not be identified to any of the other species in the genus.

*Cryophytum guerichianum* is described as having a prostrate to decumbent growth form, with angled or winged internodes, and flowers up to about 60 mm in diameter (Figure 1F). The plants observed in Hondeklip Bay were largely prostrate, but some displays of decumbence were observed. The internodes of these plants were particularly variable, with terete, angled and winged internodes, sometimes even on a single individual (Figure 1A–E). The flower size in this population was also intermediate, with flowers ranging from 26 to 48 mm, with a mean (*n* = 6) flower diameter of 38 mm. The intermediate traits displayed include internode shape, flower size and plant height. Gerbaulet and Hartmann (2017) describe *C. crystallinum* as having a prostrate growth form with terete (circular) internodes and flowers of about 15–30 mm in diameter (Figure 1G,H).

**FIGURE 2:**
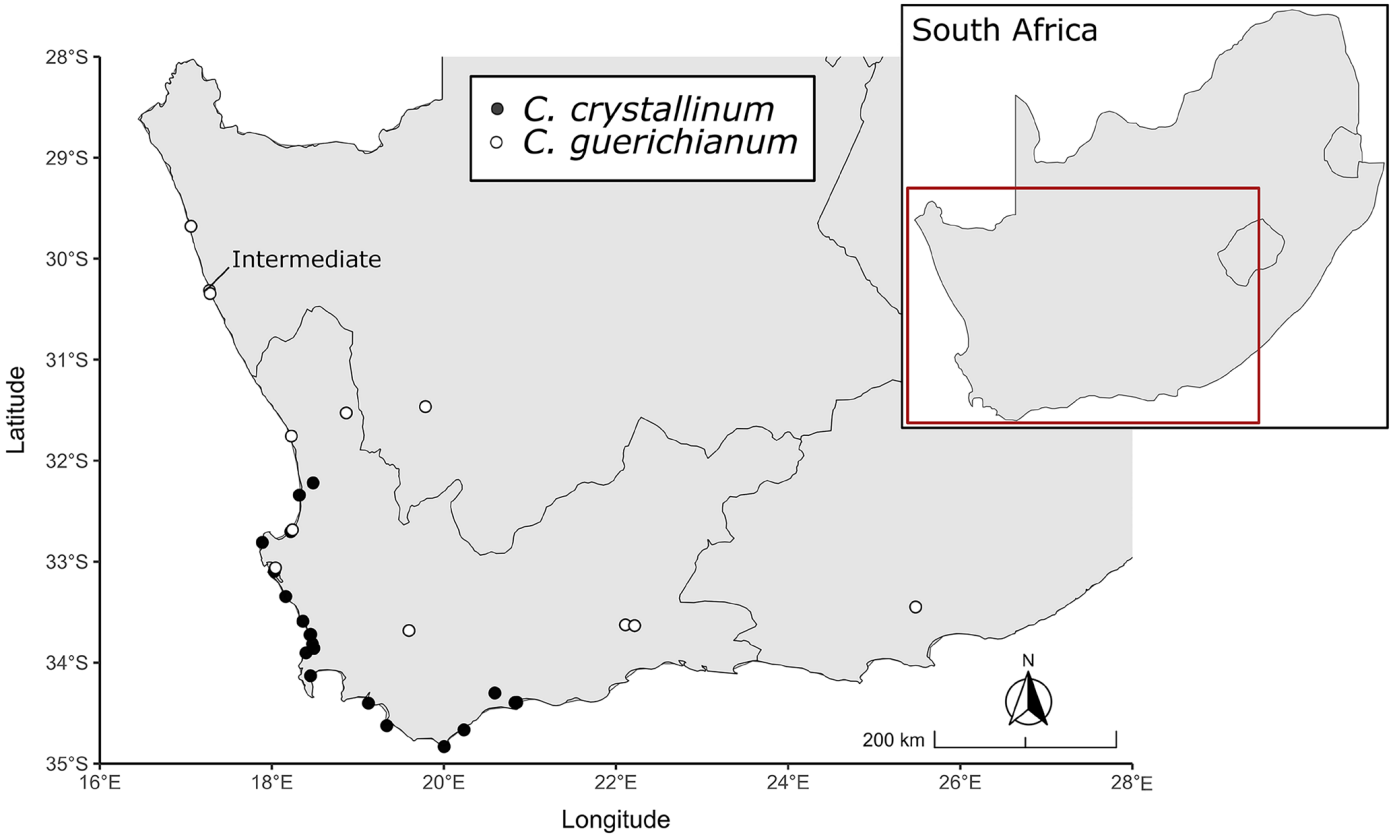
Sample collection sites of *Cryophytum crystallinum* (black dots) and *C. guerichianum* (white dots) in the native South African range. The intermediate specimens are labelled in the Northern Cape, collected in Hondeklip Bay.

In the present study, we supplemented the *C. crystallinum* RADseq dataset (*n* = 49) with additional *C. guerichianum* samples (*n* = 44) and analysed them together (File S1). All *C. guerichianum* specimens were collected in the native South African range, spanning sites in the Eastern, Western and Northern Cape Provinces (Figure 2). Our *C. crystallinum* sampling range contains some gaps when compared to the distribution presented in Gerbaulet and Hartmann (2017). Despite active searches across multiple years, we did not record any plants where the Gerbaulet and Hartmann (2017) guide reported their presence. There were also no records of the plant in those areas on iNaturalist at the time of our surveys. We established that *Cryophytum crystallinum* does not occur further east than Arniston Village and De Hoop, and despite extensive searches in the Northern Cape, we found no evidence of the plants being present in that province (Figure 2).

### 2.2 Laboratory Methods

All laboratory protocols followed those in van Steenderen et al. (2026), which applied the Adapterama III methodology (Bayona‐Vásquez et al. 2019). The ClaI and EcoRI iTru i5 and iTru i7 internal indexes, respectively, were utilised, where restriction digestion was performed using the MspI read 1 and EcoRI‐HF read 2 enzymes and the ClaI read 1 adapter dimer‐cutting enzyme. The external index pair iTru5 01 A and iTru7 101 01 was added during final library PCR preparation. Sequencing (NovaSeq X plus 10B 2 × 150 instrument; 1 lane, with 2–2.5B PE per lane) and size‐selection (525 bp ± 10% using a Blue Pippin) were outsourced to Admera Health (https://www.admerahealth.com/).

### 2.3 RADseq Bioinformatics

Bioinformatic analyses were performed on a Linux system via the Centre for High Performance Computing (CHPC) platform, hosted by the South African Council for Scientific and Industrial Research (CSIR). Following quality checks using FastQC (Andrews 2010), samples were demultiplexed using the process radtags function in Stacks (Catchen et al. 2013), with final read lengths truncated to 140 bp. Failed reads were discarded using the filter‐illumina parameter, and barcode‐dist was set to 2. Demultiplexed sample files were normalised by randomly subsampling reads using the reformat.sh script from the BBMap package (Bushnell 2014).

The reference genome for *C. crystallinum* (Sato et al. 2024) (https://www.ncbi.nlm.nih.gov/datasets/genome/GCA_030267885.1/) was used to align all demultiplexed sample fragments, which were then sorted using Bowtie2 (Langmead and Salzberg 2012) and Samtools (Li et al. 2009). The Stacks ref. map.pl. and populations functions were used to generate SNP files, which were exported to R (R Core Team 2025) for downstream processing.

SNP filtering was conducted using the SNPfiltR (DeRaad 2022) and vcfR (Knaus and Grünwald 2017) packages in R. Filtered VCF files were then converted into suitable formats for fastSTRUCTURE and SplitsTree using the dartR package (Gruber et al. 2018). SNPs were filtered to remove genotypes with a quality score < 35 (2.73% of the data) and read depth < 5 (16.2%). An additional 11.24% of heterozygous genotypes were removed based on allele balance ratio checks. SNPs with a mean depth > 100 were excluded, representing 14.77% of the data. Sample‐level missing data were filtered using a cut‐off value of 0.9. SNPs with a minor allele count (MAC) < 3 were removed (57.71% of the data). A final SNP‐level missing data filter was applied using a threshold of 0.8, which removed 72.95% of SNPs. After retaining only samples collected within the native South African range, the final filtered SNP dataset contained 96 samples and was used for clustering analyses and population‐level statistical tests.

Two separate analyses were conducted using fastSTRUCTURE (Wang 2022): one including both *C. crystallinum* and *C. guerichianum*, and another including only *C. guerichianum* and the intermediate samples. *K*‐values ranging from 1 to 10 were used to explore cluster assignments. The optimal *K*‐value was selected based on the fastSTRUCTURE test that optimised structure in the data. Additionally, a NeighbourNet diagram was constructed in SplitsTree (Huson and Bryant 2006).

Population statistics were generated using the *poppr* (poppr, private alleles) and *hierfstat* (basic.stats, allelic.richness, genet.dist and boot.ppfst) packages, with hierarchical grouping structures based on (1) SplitsTree clades and (2) collection sites. *F*_ST_ 95% confidence intervals were calculated using 10,000 bootstrap replicates in the boot.ppfst function. All data are available on the NCBI Short Read Archive (SRA) under project ID PRJNA1304995.

### 2.4 Isolation by Environment

In order to investigate the relationship between the abiotic environment and the genetic structuring of *C. crystallinum* and *C. guerichianum* populations, climate data from the WorldClim (Fick and Hijmans 2017) and SoilGRIDS (0–5 cm below the soil) (Poggio et al. 2021) databases were downloaded using the *geodata* v0.5–9 package (Hijmans et al. 2023). A subset of 12 environmental variables was selected from these to characterise site conditions for the two species (i.e., data for each ice plant's GPS record were extracted from these rasters). These included seven WorldClim bioclimatic variables: mean annual temperature (*bio 1*), maximum temperature of the warmest month (*bio 5*), minimum temperature of the coldest month (*bio 6*), and mean annual precipitation (*bio 12*), chosen for their biological relevance to the species’ ecology. Additionally, five soil‐related variables were selected: coarse fragment volume in the top 0–5 cm of soil (*cfvo*), clay content (*clay*), total nitrogen (nitrogen), organic carbon density (*ocd*) and soil organic carbon content (*soc*). Other soil properties included volumetric water content (*phh2o*), sand content (*sand*) and silt content (*silt*). These variables were chosen to capture key aspects of temperature, moisture and substrate characteristics that are likely to influence the distribution of these plants.

We assessed pairwise collinearity among all predictors using Pearson's correlation coefficients and used these results to identify redundant variables. We screened for near‐perfect correlations and for variables that provided no independent information (i.e., could be derived from others). This resulted in the exclusion of the silt percentage and one organic carbon variable. Although some moderate to strong correlations remained among the retained predictors, these were not considered problematic, as the objective of the analysis was to characterise broad abiotic patterns. Principal component analysis (PCA) is specifically designed to handle correlated variables through transformation and is therefore appropriate for use in this context.

PCAs were run on both the genetic SNP data (*glPca* function in the *adegenet* v2.1.11 R package (Jombart 2008)) and environmental variables (*prcomp* function). In order to explore which environmental variables might influence distribution patterns, a PCA biplot was generated for both species in combination using the *fviz pca biplot* function in the *factoextra* v1.0.7 package (Kassambara and Mundt 2020). The loadings for PC1 and PC2 from the SNP and environmental analyses were then merged, converted to Euclidean distance matrices, and used to run Partial Mantel tests (999 permutations) and linear models using the *partial.mantel* function in the *vegan* v2.6–4 package (Oksanen et al. 2022) and the *lm* function in the *stats* package. Partial Mantel tests were conducted for both species combined and individually, accounting for geographic distance between sites as a confounding factor. Individual species analyses excluded the intermediate (suspected hybrid) samples. Partial Mantel tests can be useful in determining whether there is a correlation between genetic and environmental distance, independent of geographic distance.

## 3 Results

### 3.1 RADseq Output

Demultiplexed samples yielded a total of 149,552,731 paired‐end reads. Following assembly, 152,851 loci were genotyped, with a mean per‐sample depth of 103.5 × ±125.4× (min = 7.6×, max = 861.4×). The mean number of sites per locus was 232.1. Average alignment success to the reference genome was 72.78% ± 15.95% across all samples (min = 13.23%, max = 93.18%). After downstream filtering in *SNPfiltR*, the final dataset contained 96 samples, with 27,587 SNPs and 8.57% missing data (File S1). The dataset included 54 *C. crystallinum* and 39 *C. guerichianum* specimens, and 3 samples that were treated as intermediates between the two species (File S1).

### 3.2 Population Structure

**FIGURE 3:**
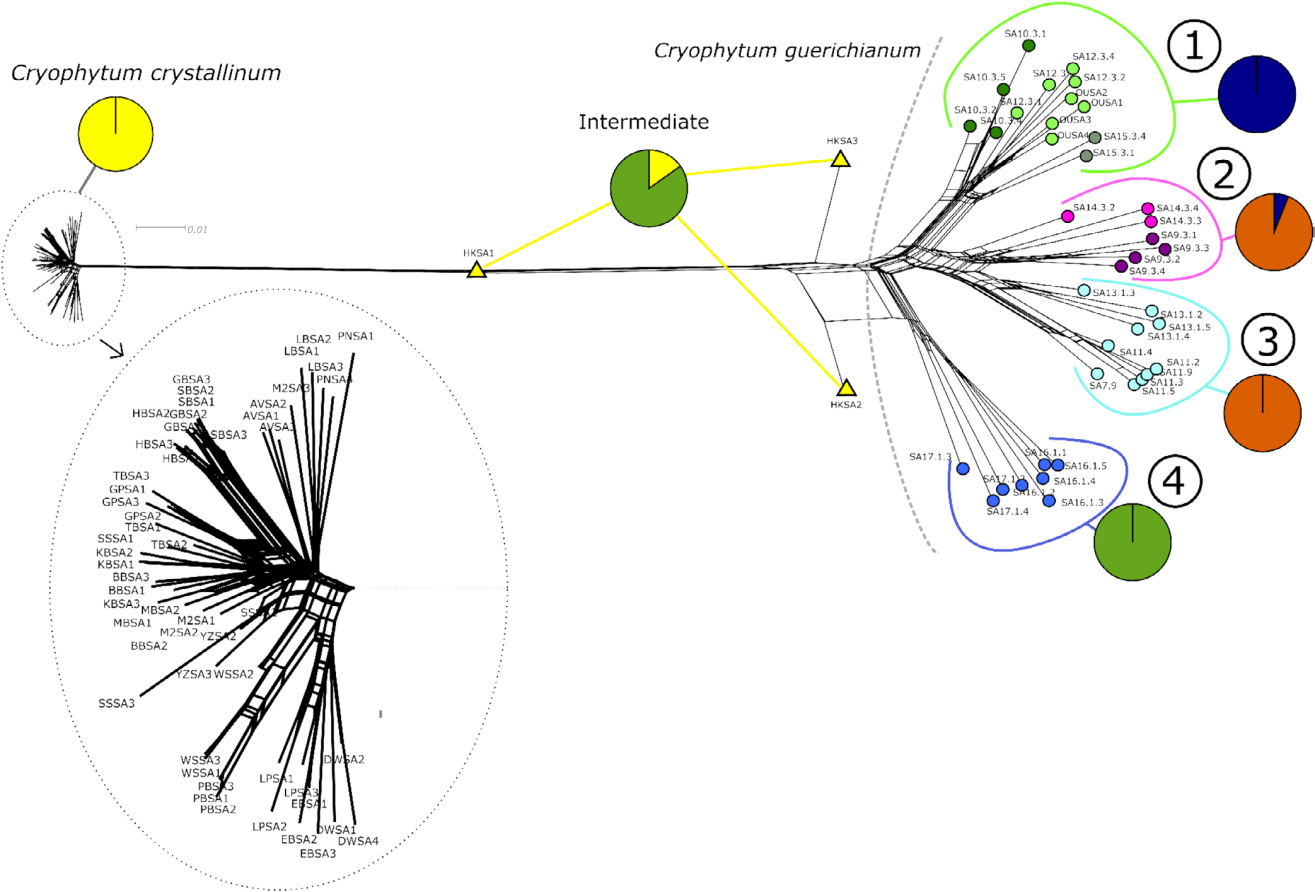
NeighbourNet SplitsTree diagram showing the separation between *Cryophytum crystallinum* and *C. guerichianum*, potential hybrids (yellow triangles), and population structuring within *C. guerichianum* (Clades 1–4). The coloured pie charts to the right of each clade are averaged fastSTRUCTURE results for *K* = 5 (see Figure 7).

**FIGURE 4:**
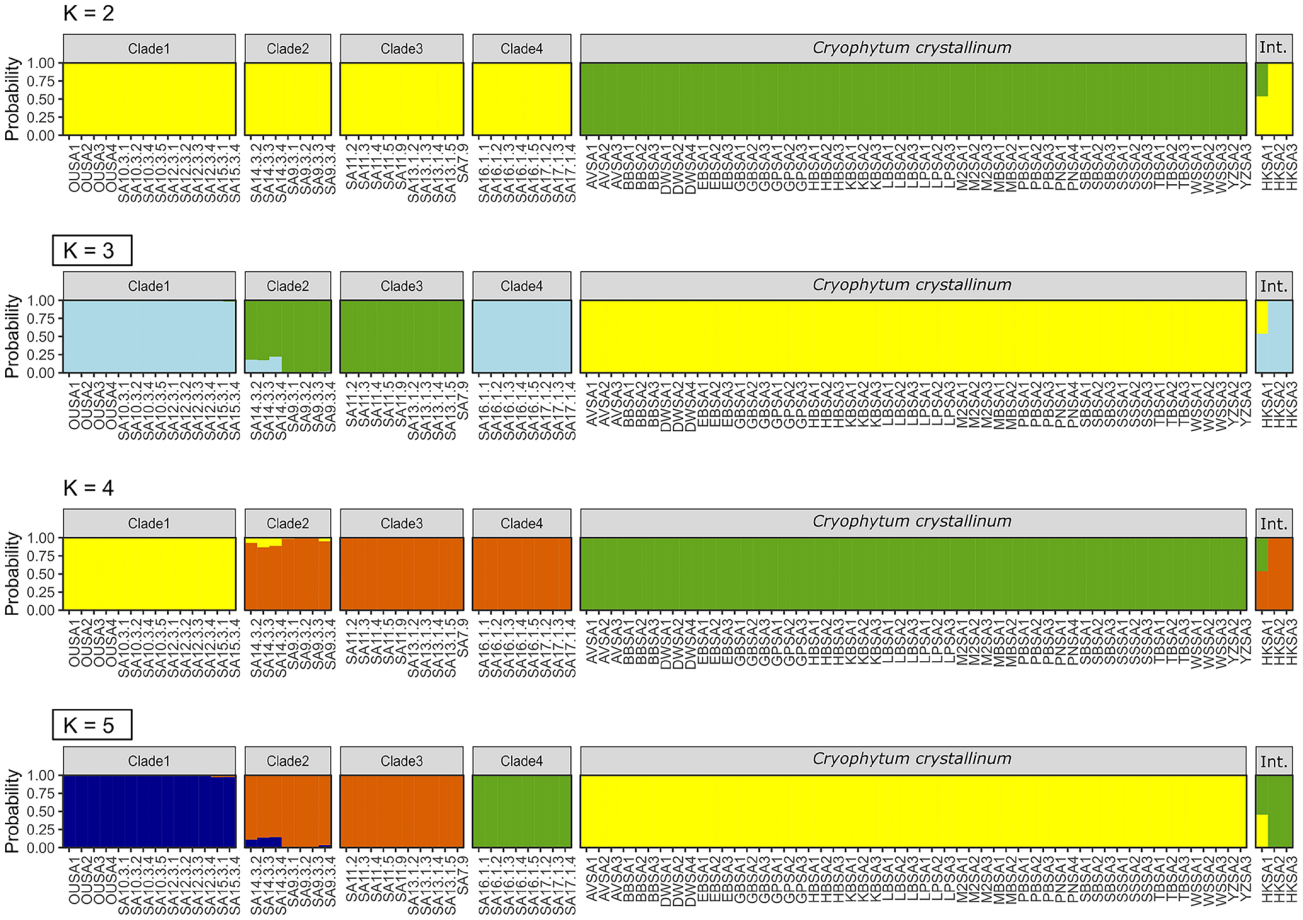
Clustering results from fastStructure analyses where both *Cryophytum crystallinum* and *C. guerichianum* were included. The optimal *K* values were *K* = 3 and *K* = 5. Clade numbers correspond to those used in Figure 7. The last panel in each diagram denotes the intermediate forms (‘Int.’).

**FIGURE 5:**
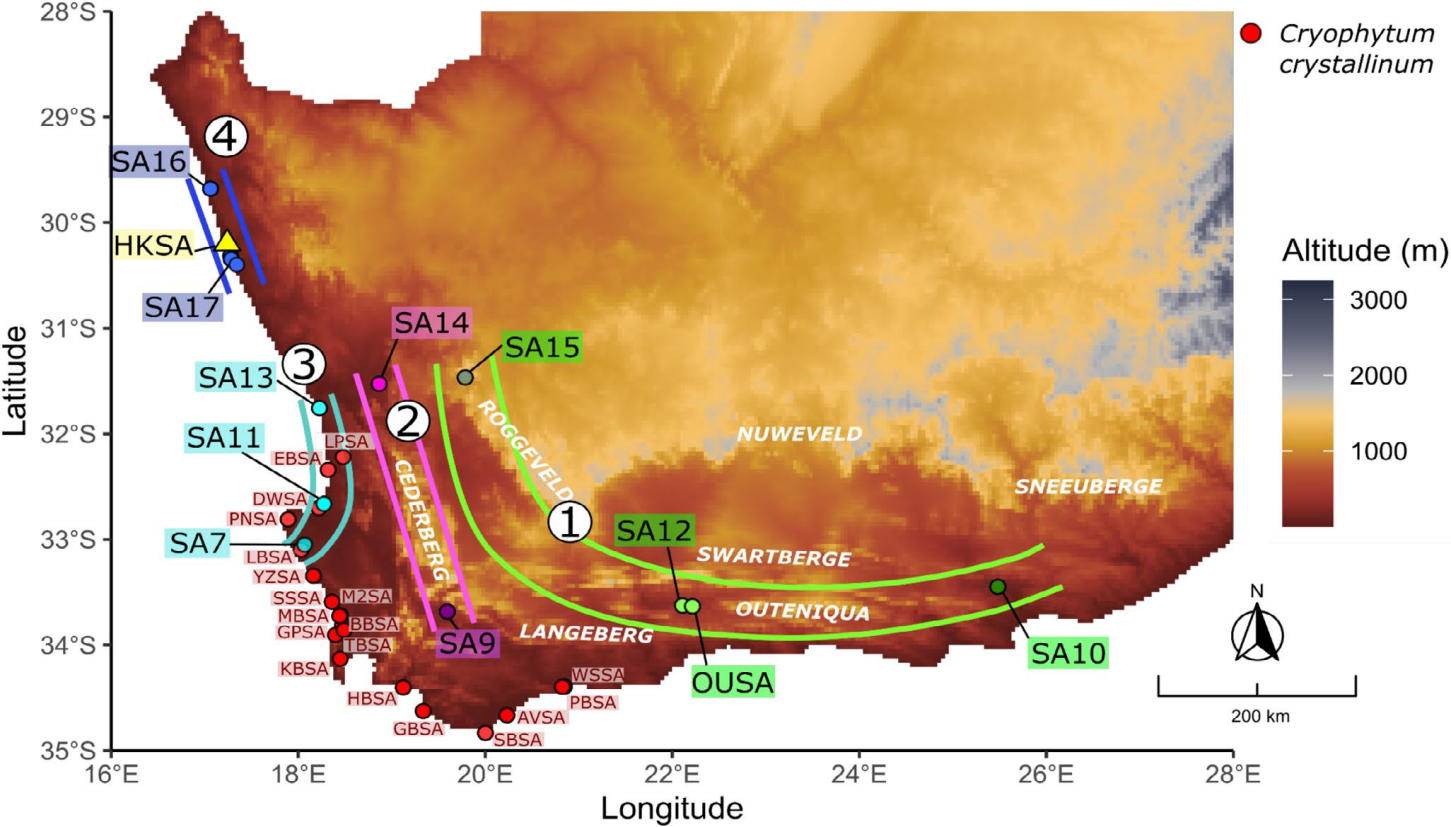
Elevation map of South Africa, showing the locations of prominent mountain ranges. The geographic distributions of Clades 1 to 4 from the SplitsTree diagram in Figure 2 are shown. *Cryophytum crystallinum* sampling sites are shown as red circles and occurred exclusively along the western and southern coastline.

The SplitsTree NeighbourNet analysis revealed a clear distinction between the *Cryophytum crystallinum* and *C. guerichianum* samples (Figure 3), a pattern that was also supported by the fastSTRUCTURE results (Figure 4). Two of the three intermediate plants from Hondeklip Bay (HKSA2 and HKSA3) clustered more closely with *C. guerichianum*, while the third (HKSA1) appeared intermediate between the two species (Figures 3 and 4). The *C. guerichianum* samples formed four distinct clades (Figure 4), corresponding to a biogeographical pattern that separated inland and coastal populations, along with an isolated group in the Northern Cape (Figure 5).

Both fastSTRUCTURE analyses reported *K* = 2 as the optimal value that explained model complexity and structure, although *K* = 3 and *K* = 4 provided further insight into *C. guerichianum* population structuring. Under *K* = 3 and *K* = 4, two of the three intermediate Hondeklip Bay samples matched Clade 4 (Figure 4). Under *K* = 4, Clade 1 and Clade 4 predominantly formed two distinct clusters, while Clade 2 and Clade 3 shared a cluster on average (Figure 4).

### 3.3 Population Statistics

At the clade level, *F*_st_ values indicated that the intermediate plants from Hondeklip Bay were most genetically similar to the *C. guerichianum* Clade 4 population and most divergent from *C. crystallinum* (Table 1). Among the four *C. guerichianum* clades, Clade 2 and Clade 3 were the most similar, while Clade 1 and Clade 3 were the most divergent (Table 1). *Cryophytum guerichianum* Clade 1 and Clade 4 had the highest number of private alleles, whereas the intermediate samples had the fewest (Table 2). Measures of allele richness (AR), observed heterozygosity (*H*_o_) and expected heterozygosity (*H*_s_) were relatively consistent across clades (Table 2). The lowest and highest inbreeding coefficients (*F*_is_) were observed in *C. guerichianum* Clade 1 and Clade 3, respectively (Table 2). A similar pattern was found for the index of association (*I*_A_) and the standardised *r*_d_. Diversity indices followed the pattern *C. guerichianum* Clade 1 > Clade 3 > Clade 4 > Clade 2, with the intermediate forms showing the lowest values (Table 2).

**TABLE 1:** Pairwise FST values between individual *Cryophytum guerichianum* populations (Clades 1–4), *C. crystallinum* as a single group, and the intermediate specimens collected in Hondeklip Bay.

	Clade1	Clade2	Clade3	Clade4	*C. crystallinum*	Intermediate
Clade1		0.32/0.34	0.4/0.42	0.38/0.4	0.87/0.87	0.32/0.34
Clade2	0.33		0.24/0.26	0.33/0.35	0.88/0.89	0.26/0.28
Clade3	0.41	0.25		0.38/0.39	0.88/0.88	0.31/0.33
Clade4	0.39	0.34	0.38		0.87/0.87	0.01/0.02
*C. crystallinum*	0.87	0.88	0.88	0.87		0.82/0.84
Int.	0.33	0.27	0.32	0.02	0.83	

*Note:* Values below the diagonal are FST values, and values above are the lower and upper confidence intervals (10,000 bootstrap repeats), separated with a forward slash (/).

**TABLE 2:** Population statistics at the clade level for *Cryophytum crystallinum* and *C. guerichianum* (Clades 1–4).

Pop	*N*	MLG	H	G	lambda	Hexp	*I*_a_	*r*_d_	*F*_is_	*F*_is_ (SE)	*H*_o_	*H*_o_ (SE)	*H*_s_	*H*_s_ (SE)	AR	AR (SE)	PA
Clade1	14	14	2.64	14.00	0.93	0.11	92.72	0.02	0.22	0.01	0.08	0.00	0.11	0.00	1.11	0.00	1326
Clade2	7	7	1.95	7.00	0.86	0.12	127.43	0.03	0.26	0.01	0.08	0.00	0.13	0.00	1.12	0.00	412
Clade3	10	10	2.30	10.00	0.90	0.11	305.07	0.06	0.29	0.01	0.07	0.00	0.12	0.00	1.11	0.00	756
Clade4	8	8	2.08	8.00	0.88	0.14	30.22	0.01	0.23	0.01	0.10	0.00	0.14	0.00	1.14	0.00	794
*C. cryst*.	54	54	3.99	54.00	0.98	0.03	72.00	0.04	0.77	0.01	0.01	0.00	0.03	0.00	1.03	0.00	3130
Intermediate	3	3	1.10	3.00	0.67	0.14	1.92	0.00	0.06	0.01	0.12	0.00	0.15	0.00	1.14	0.00	8

Abbreviations: AR, allele richness; *F*_is_, fixation index (inbreeding coefficient); G, Stoddard and Taylor's Index; H, Shannon‐Weiner Diversity Index; Hexp, Nei's gene diversity; *H*_o_, Observed Heterozygosity; *H*_s_, Expected Heterozygosity; *I*_a_, Index of Association; lambda, Simpson's Index; MLG, Multilocus Genotypes; eMLG, expected number of MLGs; *N*, Number of individuals; PA, private alleles; *r*_d_, Standardised Index of Association.

At the site level, *C. guerichianum* SA9, SA16, SA17, SA13 and SA14 had the highest number of private alleles, while SA7 had the lowest (Table 3). Sites SA16 and SA17 also showed the highest inbreeding coefficients, whereas SA10 was the only site with a negative value, suggesting potential outbreeding. Sites SA11 and SA16 showed the highest diversity indices, in contrast to SA7 and SA15, which had the lowest.

**TABLE 3:** Within‐population statistics at the site level for *Cryophytum crystallinum* and *C. guerichianum* specimens.

Pop	*N*	MLG	H	G	lambda	Hexp	*I*_a_	*r*barD	*F*_is_	*F*_is_ (SE)	*H*_o_	*H*_o_ (SE)	*H*_s_	*H*_s_ (SE)	AR	AR (SE)	PA
AVSA	3	3	1.10	3.00	0.67	0.02	10.80	0.02	0.43	0.02	0.01	0.00	0.02	0.00	1.02	0.00	4
BBSA	3	3	1.10	3.00	0.67	0.01	0.42	0.00	0.75	0.03	0.00	0.00	0.01	0.00	1.01	0.00	0
DWSA	3	3	1.10	3.00	0.67	0.02	86.21	0.19	0.46	0.02	0.01	0.00	0.02	0.00	1.02	0.00	25
EBSA	3	3	1.10	3.00	0.67	0.02	107.10	0.25	0.60	0.02	0.01	0.00	0.02	0.00	1.02	0.00	40
GBSA	3	3	1.10	3.00	0.67	0.00	19.82	0.21	0.17	0.06	0.00	0.00	0.00	0.00	1.00	0.00	2
GPSA	3	3	1.10	3.00	0.67	0.01	121.24	0.46	0.57	0.03	0.00	0.00	0.01	0.00	1.01	0.00	3
HBSA	3	3	1.10	3.00	0.67	0.00	29.22	0.81	0.07	0.11	0.00	0.00	0.00	0.00	1.00	0.00	5
HKSA	3	3	1.10	3.00	0.67	0.14	1.92	0.00	0.06	0.01	0.12	0.00	0.15	0.00	1.14	0.00	8
KBSA	3	3	1.10	3.00	0.67	0.00	0.00	0.00	−0.75	0.12	0.00	0.00	0.00	0.00	1.00	0.00	2
LBSA	3	3	1.10	3.00	0.67	0.02	0.03	0.00	0.51	0.02	0.01	0.00	0.03	0.00	1.02	0.00	9
LPSA	3	3	1.10	3.00	0.67	0.02	34.98	0.08	0.29	0.02	0.01	0.00	0.02	0.00	1.02	0.00	16
M2SA	3	3	1.10	3.00	0.67	0.02	79.40	0.16	0.32	0.02	0.01	0.00	0.02	0.00	1.02	0.00	0
MBSA	2	2	0.69	2.00	0.50	0.01			−0.02	0.03	0.01	0.00	0.01	0.00	1.01	0.00	0
OUSA	4	4	1.39	4.00	0.75	0.09	110.54	0.04	0.05	0.01	0.08	0.00	0.10	0.00	1.09	0.00	110
PBSA	3	3	1.10	3.00	0.67	0.00	−0.40	−0.06	−0.39	0.19	0.00	0.00	0.00	0.00	1.00	0.00	8
PNSA	2	2	0.69	2.00	0.50	0.02			0.40	0.03	0.01	0.00	0.03	0.00	1.02	0.00	7
SA10	4	4	1.39	4.00	0.75	0.08	13.97	0.01	−0.15	0.01	0.09	0.00	0.08	0.00	1.08	0.00	164
SA11	5	5	1.61	5.00	0.80	0.07	95.29	0.04	0.10	0.01	0.06	0.00	0.07	0.00	1.07	0.00	225
SA12	4	4	1.39	4.00	0.75	0.10	36.93	0.01	0.09	0.01	0.08	0.00	0.10	0.00	1.10	0.00	67
SA13	4	4	1.39	4.00	0.75	0.11	33.75	0.01	0.11	0.01	0.09	0.00	0.12	0.00	1.11	0.00	143
SA14	3	3	1.10	3.00	0.67	0.11	58.42	0.02	0.14	0.01	0.09	0.00	0.12	0.00	1.11	0.00	64
SA15	2	2	0.69	2.00	0.50	0.08			0.06	0.01	0.07	0.00	0.09	0.00	1.08	0.00	23
SA16	5	5	1.61	5.00	0.80	0.13	2.16	0.00	0.16	0.01	0.10	0.00	0.13	0.00	1.13	0.00	289
SA17	3	3	1.10	3.00	0.67	0.13	38.56	0.01	0.15	0.01	0.10	0.00	0.14	0.00	1.13	0.00	54
SA7	1	1	0.00	1.00	0.00	0.05					0.05	0.00			1.05	0.00	1
SA9	4	4	1.39	4.00	0.75	0.10	44.61	0.02	0.11	0.01	0.08	0.00	0.10	0.00	1.10	0.00	221
SBSA	3	3	1.10	3.00	0.67	0.00	0.79	0.22	−0.62	0.16	0.00	0.00	0.00	0.00	1.00	0.00	5
SSSA	3	3	1.10	3.00	0.67	0.02	150.95	0.33	0.80	0.02	0.00	0.00	0.03	0.00	1.02	0.00	3
TBSA	3	3	1.10	3.00	0.67	0.02	3.16	0.01	0.49	0.02	0.01	0.00	0.02	0.00	1.02	0.00	6
WSSA	3	3	1.10	3.00	0.67	0.00	22.91	0.36	0.12	0.08	0.00	0.00	0.00	0.00	1.00	0.00	14
YZSA	2	2	0.69	2.00	0.50	0.02			−0.17	0.04	0.02	0.00	0.02	0.00	1.02	0.00	1

*Note:* Missing values resulted from only one representative sample per group and could not be computed. Refer to File S1 for full sample details.

Abbreviations: AR, allele richness; *F*_is_, fixation index (inbreeding coefficient); G, Stoddard and Taylor's Index; H, Shannon‐Weiner Diversity Index; Hexp, Nei's gene diversity; *H*_o_, Observed Heterozygosity; *H*_s_, Expected Heterozygosity; *I*_a_, Index of Association; lambda, Simpson's Index; MLG, Multilocus Genotypes; eMLG, expected number of MLGs; *N*, Number of individuals; PA, private alleles; *r*_d_, Standardised Index of Association.

### 3.4 Isolation by Environment

The environmental variables with the top three highest loadings on PC1 were soil organic carbon, maximum temperature in the warmest month (bio 5), and soil pH. For PC2 these were coarse fragments and sand content (Table 4). Cumulatively, the first two PCs contributed 72% to the variation in the data.

**TABLE 4:** Principal Component Analysis (PCA) loadings for the first four principal components (PC1–PC4) derived from environmental variables (WorldClim and soil data).

	PC1	PC2	PC3	PC4
Mean Temp (Bio1)	−0.27	0.29	0.24	−0.71
Max Temp (Bio5)	−0.39	0.16	0.30	−0.06
Min Temp (Bio6)	0.34	−0.17	−0.04	−0.61
Annual Precip (Bio12)	0.36	0.33	0.11	0.10
Coarse Fragments	0.01	−0.46	−0.58	−0.28
Clay %	−0.28	0.37	−0.35	−0.09
Soil N	0.33	0.36	−0.21	0.03
Soil pH	−0.37	−0.25	−0.14	0.15
Sand %	0.17	−0.45	0.56	−0.04
Soil Organic C	0.42	0.13	−0.03	−0.05

*Note:* Loadings represent the contribution of each variable to each component.

**FIGURE 6:**
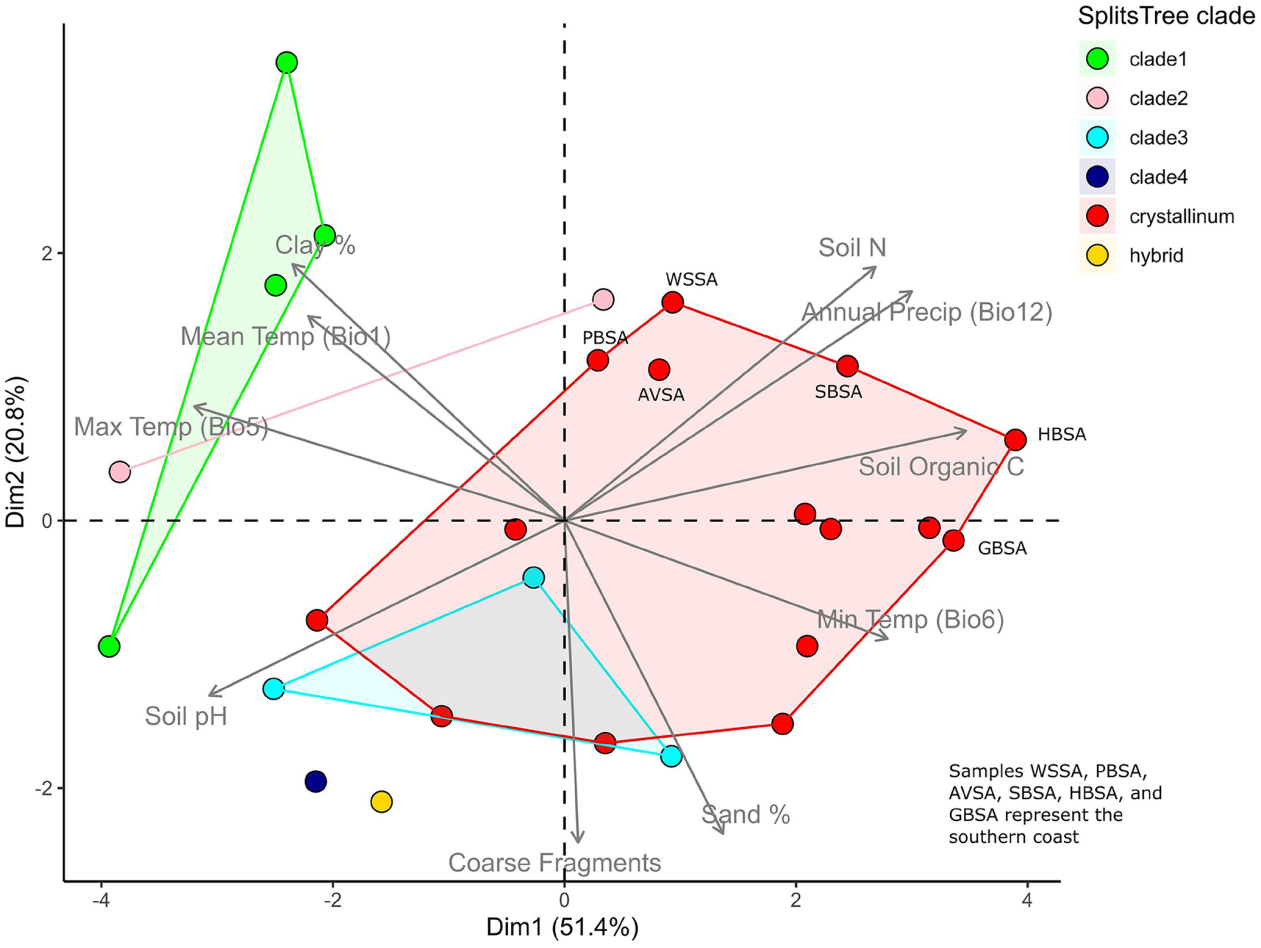
Principal Components Analysis (PCA) of the selected WorldClim and soil variables, showing PC1 and PC2 with overlaid vector lines. Points are coloured according to the SplitsTree clades in Figure 2.

The PCA biplot showed that the inland *C. guerichianum* population, Clade 1, was distinct in its environmental profile (Figure 6), differing from the other populations and from that of *C. crystallinum* . This environmental difference was driven by greater soil pH, clay content, and temperature, and lower rainfall, soil nitrogen, organic carbon and sand content (Figure 6; Table 4). *Cryophytum guerichianum* (Clade 2) showed an intermediate profile between Clade 1 and the remaining populations and *C. crystallinum* , while *C. guerichianum* Clade 3 overlapped with *C. crystallinum* (Figure 6). *Cryophytum guerichianum* Clade 4 and the hybrids appeared as non‐overlapping separate groups.

**FIGURE 7:**
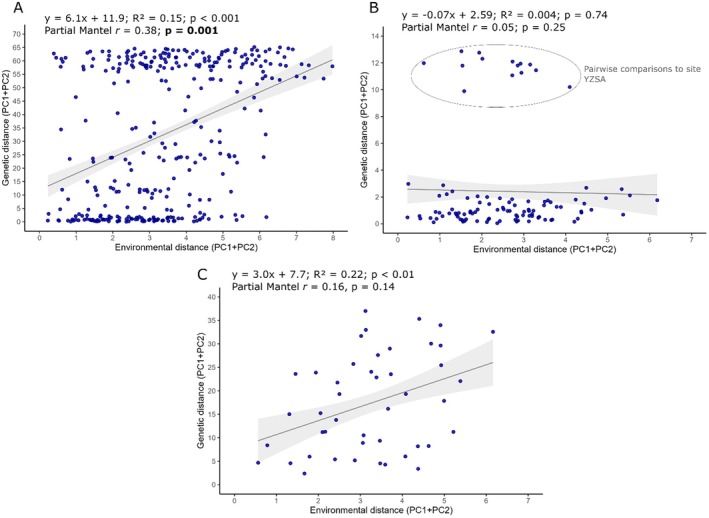
Pairwise comparison of environmental and genetic distances for (A) *Cryophytum crystallinum* and *C. guerichianum* combined, (B) *Cryophytum crystallinum*, and (C) *C. guerichianum*. Each point represents a pair of sites. Shading around the linear regression line indicates the 95% confidence interval, and the results of a Partial Mantel test, accounting for geographic distance as a confounding factor, are printed onto each graph. The circled points in (B) are all the pairwise comparisons to the Yzerfontein site (YZSA).

There was some evidence of differentiation along the PC2 axis for *C. crystallinum* , where samples from the south coast were characterised by higher soil nitrogen, rainfall, minimum temperatures, sand content, and organic carbon; and lower soil pH, mean and maximum temperatures, and clay content (Table 4; Figure 6). The Partial Mantel tests, accounting for geographic distance as a confounding factor, supported significant correlations between environmental and genetic distance when analysed for both species combined (Figure 7A–C). There was not, however, a significant relationship found when *C. crystallinum* and *C. guerichianum* were analysed alone. The Mantel plot for *C. crystallinum* in Figure 7B comprised a cluster of points that scored higher on the genetic distance axis. Upon closer inspection, these were all pairwise comparisons to the site in Yzerfontein (YZSA). We ran the Partial Mantel test a second time with these points excluded, which again revealed a non‐significant result (Mantel *r* statistic = 0.19, *p* = 0.12), although the regression line now showed a significant positive relationship (*y* = 0.09 + 0.77, *R* ^2^ = 0.02, *p* = 0.03).

## 4 Discussion

We found evidence of at least three genetically distinct *Cryophytum guerichianum* populations: an inland, intermediate and coastal group (Figure 5). These populations broadly correspond to regions separated by the Cederberg and Langeberg mountain ranges within the Cape Fold Belt, suggesting that topographic barriers may have limited gene flow between them and contributed to historical vicariance. The inland clade was the most environmentally distinct, occurring in warmer and drier regions, consistent with a climatic gradient across the study area. Although this clade was also associated with differences in soil properties (e.g., higher pH, greater clay content, and lower nitrogen, organic carbon and sand content) (Figure 6), these variables were sourced from modelled (i.e., interpolated) datasets and likely reflect broad‐scale environmental variation rather than direct drivers of genetic differentiation. As such, we interpret soil associations with caution, and consider landscape topography, potentially in combination with climatic gradients, as the more likely explanation for the observed patterns.

This pattern provides some support for the Isolation by Environment (IBE) hypothesis (Figure 7), suggesting that genetic differentiation may be associated with environmental gradients, particularly climatic variation (Lee and Mitchell‐Olds 2011; Wu et al. 2015; Jiang et al. 2019; Musker et al. 2021; Schmidt et al. 2025; Yang et al. 2025). A comparable pattern was reported in *Lithops ruschiorum*, a succulent species endemic to southern Africa. Loots et al. (2019) found evidence of isolation by distance (IBD) among populations in the Namib Desert biome. They linked population structure to differing moisture patterns, specifically fog‐derived precipitation in coastal populations versus rainfall in inland populations. They further proposed that the relatively low genetic differentiation observed among some populations may result from recent fragmentation from a historically larger, more continuous population.

In another study, Musker et al. 2021 investigated genetic differentiation in two co‐occurring Aizoaceae species, *Ruschia burtoniae* and *Conophytum calculus*, in the Succulent Karoo Knersvlakte region of South Africa. They found no evidence of gene flow in *R. burtoniae*, whereas *C. calculus* showed high admixture and a pattern consistent with IBD. These contrasting patterns were associated with differences in edaphic niche, primarily driven by variation in soil pH across the quartz fields in the region.

Focusing on the Knersvlakte and closely aligned with our study system, Schmidt et al. (2025) analysed population genetic structure in the two species of the genus *Oophytum*, *O. nanum* and *O. oviforme* , which co‐occur across a patchy distribution in the region. They identified three distinct metapopulations across the landscape and detected two genetic groups within *O. nanum* . These spatial genetic patterns were associated with environmental variables, with humidity as a potential driver of genetic isolation. In line with these findings, recurrent drought events may similarly have contributed to reduced genetic diversity in some *Cryophytum* populations in our study, likely through founder effects and genetic drift. Additionally, consistent with our results, Schmidt et al. (2025) reported evidence of hybridisation, showing that populations morphologically identified as *O. oviforme* were in fact *O. nanum* hybrids, likely resulting from multiple rounds of hybridisation and backcrossing.

Also in the Knersvlakte, Ellis et al. (2006) reported strong spatial genetic structuring in *Argyroderma*, with divergence among geographically isolated populations associated with distinct edaphic microhabitats. This differentiation was also linked to shifts in flowering phenology. At a broader spatial scale, Ellis et al. (2007) found that genetic structure corresponded with drainage basin boundaries, indicating limited gene flow between basins. Similarly, Boucher et al. (2024) found that reproductive isolation in *Argyroderma* is likely maintained by pre‐mating barriers, including fine‐scale geographic isolation and habitat differentiation associated with edaphic variation.

In our results, the inland *C. guerichianum* clade showed the greatest genetic diversity and the highest number of private alleles, suggesting that it may represent an ancestral lineage. These inland populations may have been buffered from sea‐level change during glacial cycles (Compton 2016), potentially acting as source populations for recolonisation when the continental shelf was re‐exposed during periods of lower sea levels.

Numerous other studies have linked allopatric speciation in the Cape Floristic Region (CFR) to topography, climate and edaphic variation (Cowling and Lombard 2002; Cowling et al. 2009; Britton et al. 2014; Myburgh and Daniels 2022; Tolley et al. 2022; Daniels and Barnes 2025). Our findings therefore add to growing evidence that the unique landscape configuration and Quaternary history of the CFR have been key drivers of species radiations. We acknowledge, however, that the soil variables obtained from the SoilGrids database are spatially interpolated, model‐based estimates at a resolution of 250 m and may not capture fine‐scale variation relevant to patchily distributed plant populations (Poggio et al. 2021). While our analyses include comparisons at the level of individual populations, the associated environmental data represent broader‐scale conditions rather than local microhabitats. As such, the patterns identified here are best interpreted as reflecting regional environmental gradients, and any inferences linking genetic structure to abiotic conditions at individual sites, particularly soil properties, should be treated with caution.

There was some evidence of genetic differentiation within *C. crystallinum* (Table 2), but the present results suggest that this pattern is likely driven by factors other than the abiotic environmental variables considered here. van Steenderen et al. (2026) found that *C. crystallinum* populations in the northern regions of Leipoldtville, Elands Bay and Paternoster were genetically similar to populations on the south coast, despite being separated by three genetically distinct populations occurring between them. Although the Yzerfontein site in the Western Cape Province was represented by only two *C. crystallinum* individuals, the partial Mantel analysis showed that this site had a relatively high pairwise genetic differentiation from the other populations (Figure 7B). While this pattern does not mean that within‐population genetic diversity is higher, it suggests that the sampled individuals are genetically distinct relative to the other populations. However, given the extremely small sample size for the site, this result should be interpreted with caution. If this observation is indeed a real biological signal, this pattern could suggest local isolation and genetic drift, or reduced gene flow with other populations.

As discussed above for *C. guerichianum*, historical climatic fluctuations (Midgley and Roberts 2001; Chase et al. 2019) and associated sea‐level changes may have influenced patterns of range expansion and contraction in the coastal *C. crystallinum* populations (Toms et al. 2014; Ramos‐Fregonezi et al. 2015; Parvizi et al. 2022). During periods of lower sea levels, the exposed continental shelf may have facilitated range expansion and increased connectivity, whereas subsequent sea‐level rise could have fragmented these coastal populations and promoted isolation. The *C. crystallinum* populations at either end of the distribution (northern vs. south‐eastern coasts in the Western Cape Province) may have become isolated in this way, with founder populations subsequently recolonising the intervening areas and giving rise to distinct genetic clusters.

Seed dispersal via water‐mediated mechanisms may also have contributed to gene flow between coastal populations. In Aizoaceae, dispersal is primarily driven by ombrohydrochory (rain‐operated seed ejection; Parolin 2006), which facilitates short‐distance movement during rainfall events. Surface runoff and coastal hydrological connectivity may further contribute to dispersal along the coastline. The lower genetic differentiation among *C. crystallinum* populations, relative to *C. guerichianum*, may therefore indicate increased connectivity among coastal populations. Silva‐Arias et al. (2021) reported a similar pattern in a population genetic study of *Calibrachoa heterophylla* (Solanaceae), where coastal populations showed reduced genetic differentiation linked to landscape features such as wind corridors and the timing of colonisation events.

The intermediate forms found in Hondeklip Bay were genetically more similar, on average, to the *C. guerichianum* population in the surrounding area (Table 1). Only one of the three samples showed a partial genetic match to *C. crystallinum* . This finding may indicate some degree of introgression (i.e., hybridisation followed by repeated backcrossing) between the two species, resulting in morphologically intermediate individuals that are more genetically aligned with *C. guerichianum* (i.e., asymmetric introgression). Several studies have reported similar asymmetric introgression patterns in plants, including in oaks (Peñaloza‐Ramírez et al. 2010), mulberries (Burgess et al. 2005), magnolias (Muranishi et al. 2013) and spruces (de Lafontaine and Bousquet 2017). Although our sample size for these hybrids was low (*n* = 3), the results provide some evidence of a potential hybridisation zone along the South African west coast. It remains unclear, however, why hybrids have not been detected in areas where the two species occur in sympatry, namely between Langebaan and Leipoldtville. It is possible that hybridisation is restricted to certain populations due to genetic incompatibilities, resulting in a mosaic pattern of hybridisation (Kenney and Sweigart 2016).

Morphological traits characteristic of Mesembryanthemoideae, such as epidermal bladder cells and variation in stem architecture, likely play important roles in adaptation to arid and saline environments. While these traits were not quantified in the present study, they may be associated with the environmental gradients identified here and could contribute to local adaptation among populations. Future research integrating morphological and genomic data would be valuable for disentangling the extent to which phenotypic variation is driven by genetic differentiation.

## 5 Conclusion

The findings of the present study suggest that a combination of historical landscape changes along the South African coastline, environmental variation and dispersal may have shaped the genetic structuring observed within *C. crystallinum* and *C. guerichianum*. The evidence of asymmetric introgression between the two species at Hondeklip Bay further suggested that hybridisation may only be occurring between compatible populations, and that some sympatric populations are not able to interbreed. These results add to the growing knowledge of biodiversity in the Cape region, and how climate and topography have shaped the evolutionary pathways of the unique species found there.

Understanding these historical patterns can help explain how genetically distinct populations arise within different parts of the native range, and why potential biocontrol agents may respond differently to them (Manrique et al. 2008; Mukwevho et al. 2017). Importantly, geographic distance from the source population alone should not determine where biocontrol agents are collected, as population structure in the native range can be more complex. In the current study, for example, if plant source populations were matched to the South African West Coast, suitable agents could be collected from either end of the native distribution, but not from intermediate regions. Understanding population dynamics and evolutionary history therefore helps to identify appropriate source locations for biocontrol agents.

## Author Contributions

**Clarke J. M. van Steenderen:** conceptualization (equal), data curation (equal), formal analysis (lead), investigation (lead), methodology (lead), software (lead), validation (lead), visualization (lead), writing – original draft (lead), writing – review and editing (lead). **Emma Sandenbergh:** conceptualization (equal), data curation (equal), investigation (equal), writing – review and editing (equal). **Iain D. Paterson:** conceptualization (equal), funding acquisition (lead), investigation (equal), project administration (lead), resources (lead), supervision (lead), writing – review and editing (equal).

## Funding

This work was supported by U.S. Department of Defense. Centre for Biological Control at Rhodes University, U.S. Navy, U.S. Department of Agriculture.

National Research Foundation.

## Conflicts of Interest

The authors declare no conflicts of interest.

## Supporting information


**File S1:** Information sheet for the 96 RADseq samples used in the present population genetics study. Represented are 49 Cryophytum crystallinum, 44 C. guerichianum and three hybrid samples collected in South Africa.

## Data Availability

RADseq data files are available on the SRA database (https://www.ncbi.nlm.nih.gov/bioproject/?term=PRJNA1304995) under Project ID PRJNA1304995, or upon request from the CBC. All the related R code and input data are available on a public GitHub repository: https://github.com/clarkevansteenderen/cryophytum_biogeography/tree/main which is linked to a permanent Zenodo doi: 10.5281/zenodo.19438438.
